# The impact of sagittal balance on clinical results after posterior interbody fusion for patients with degenerative spondylolisthesis: A Pilot study

**DOI:** 10.1186/1471-2474-12-69

**Published:** 2011-04-05

**Authors:** Mi Kyung Kim, Sun-Ho Lee, Eun-Sang Kim, Whan Eoh, Sung-Soo Chung, Chong-Suh Lee

**Affiliations:** 1 Department of Neurosurgery, Spine center, Samsung Medical Center, Sungkyunkwan University, School of Medicine, Seoul, 135-710, Republic of Korea; 2 School of Medicine, Konkuk University Seoul, 143-701, Republic of Korea; 3 Department of Orthopedic Surgery, Spine center, Samsung Medical Center, Sungkyunkwan University, School of Medicine, Seoul, 135-710, Republic of Korea

## Abstract

**Background:**

Comparatively little is known about the relation between the sagittal vertical axis and clinical outcome in cases of degenerative lumbar spondylolisthesis. The objective of this study was to determine whether lumbar sagittal balance affects clinical outcomes after posterior interbody fusion. This series suggests that consideration of sagittal balance during posterior interbody fusion for degenerative spondylolisthesis can yield high levels of patient satisfaction and restore spinal balance

**Methods:**

A retrospective study of clinical outcomes and a radiological review was performed on 18 patients with one or two level degenerative spondylolisthesis. Patients were divided into two groups: the patients without improvement in pelvic tilt, postoperatively (Group A; n = 10) and the patients with improvement in pelvic tilt postoperatively (Group B; n = 8). Pre- and postoperative clinical outcome surveys were administered to determine Visual Analogue Pain Scores (VAS) and Oswestry disability index (ODI). In addition, we evaluated full spine radiographic films for pelvic tilt (PT), sacral slope (SS), pelvic incidence (PI), thoracic kyphosis (TK), lumbar lordosis (LL), sacrofemoral distance (SFD), and sacro C7 plumb line distance (SC7D)

**Results:**

All 18 patients underwent surgery principally for the relief of radicular leg pain and back pain. In groups A and B, mean preoperative VAS were 6.85 and 6.81, respectively, and these improved to 3.20 and 1.63 at last follow-up. Mean preoperative ODI were 43.2 and 50.4, respectively, and these improved to 23.6 and 18.9 at last follow-up. In spinopelvic parameters, no significant difference was found between preoperative and follow up variables except PT in Group A. However, significant difference was found between the preoperative and follows up values of PT, SS, TK, LL, and SFD/SC7D in Group B. Between parameters of group A and B, there is borderline significance on preoperative PT, preoperative LL and last follow up SS.

Correlation analysis revealed the VAS improvements in Group A were significantly related to postoperative lumbar lordosis (Pearson's coefficient = -0.829; p = 0.003). Similarly, ODI improvements were also associated with postoperative lumbar lordosis (Pearson's coefficient = -0.700; p = 0.024). However, in Group B, VAS and ODI improvements were not found to be related to postoperative lumbar lordosis and to spinopelvic parameters.

**Conclusion:**

In the current series, patients improving PT after fusion were found to achieve good clinical outcomes in degenerative spondylolisthesis. Overall, our findings show that it is important to quantify sagittal spinopelvic parameters and promote sagittal balance when performing lumbar fusion for degenerative spondylolisthesis.

## Background

The clinical outcomes of spinal fusion in degenerative spondylolisthesis are influenced by a variety of pathophysiologic factors, such as, the recurrence of spinal canal stenosis, instability, lumbar kyphosis, nonunion, and the disturbance of adjacent segments [1,2]. Although satisfactory clinical outcomes have been reported for a variety of surgical techniques, the optimal management of degenerative spondylolisthesis remains controversial. Recently, laboratory and clinical evidence has indicated that if fusion surgery is undertaken, improved short- and long-term outcomes can be achieved by correcting any sagittal deformity present [3-5].

Some studies have attempted to correlate spinopelvic parameters with health related quality of life (HRQOL) or pain measures in order to provide some insight during surgical planning for isthmic spondylolisthesis [6,7]. These studies identified key radiographic parameters that are correlated with patient pain and disability, and found that pelvic tilt (PT) is related to HRQOL[8]. However, these studies were not without limitations, and evidence supporting this relationship remains limited and it has not been determined whether sagittal vertical axis influences clinical outcomes in degenerative spondylolisthesis [9-11].

The purpose of this study was to explore the relationships between pelvic tilt and other spinopelvic parameters and with clinical outcomes after spinal fusion for degenerative spondylolisthesis. In addition, we attempted to determine whether specific critical values of spinopelvic parameters can predict poor HRQOL, and thus, aid surgical planning.

## Methods

### Patient characteristics

We retrospectively reviewed 220 patients who underwent surgery for degenerative spondylolisthesis from July 2003 to June 2008 at our institute. All patients were operated on by four senior surgeons (KES, WE, CSS and LJS). Eighteen of these patients were selected for this study by applying the following criteria: 1) one or two-level degenerative lumbar spondylolisthesis; 2) treatment by posterior interbody fusion; 3) patients showing radiological solid fusion on follow up computed tomography (CT); and 4) a minimum clinical and radiologic follow-up of 24 months. The following exclusion criteria were applied: 1) more than three-level fusion; 2) a history of a previous spinal operation; and 3) the presence of severe systemic disease, a vertebral fracture, or osteoporosis. All patients visited our outpatient department in June 2010, and a trained nurse collected follow-up clinical data. The medical records of patients were reviewed, along radiographic studies that included preoperative and postoperative radiographs, computed tomography (CT) scans, and magnetic resonance images.

Average patient age was 60.8 years (48-72 years), and there were 8 men and 10 women. Mean symptom duration before surgery was 2.3 years (3 months - 10 years), and patients were observed for an average of 43.2 months (24-84 months). 14 patients underwent single-level fusions, of which 12 were at L4-L5 and 2 at L3-4. There were four two-level fusions, one at L4-L5-S1 and three at L3-L4-L5. Demographic data are summarized in table 1. The study was authorized by the institutional review board of the Samsung medical center. Informed consent was obtained from all patients in accordance with the institutional review board at our institution.

**Table 1:** Summary of patient demographic and preoperative clinical characteristics

Characteristic	Case	
	
	Group A (n = 10)	Group B (n = 8)
Sex (M:F)	3:7	5:3
Mean age in yrs (range)	61.0 (48-72)	60.5 (48-69)
Spinal level		
L3-4	1	1
L4-5	7	5
L3-4-5	2	1
L4-5-S1	0	1

### Clinical and radiological evaluations

All patients were examined clinically and radiographically before and after surgery at the following times: immediately after surgery and at 3, 6, and 12 months and annually thereafter. At clinical evaluations, all patients completed the Visual Analogue Pain Score (VAS) for back pain and the Oswestry disability index (ODI) questionnaire in order to assess HRQOLs [12]. Clinical outcome was evaluated with improvement in VAS and in ODI. Preoperative and final follow up data were assessed using clinical charts and operative reports.

**Figure 1:**
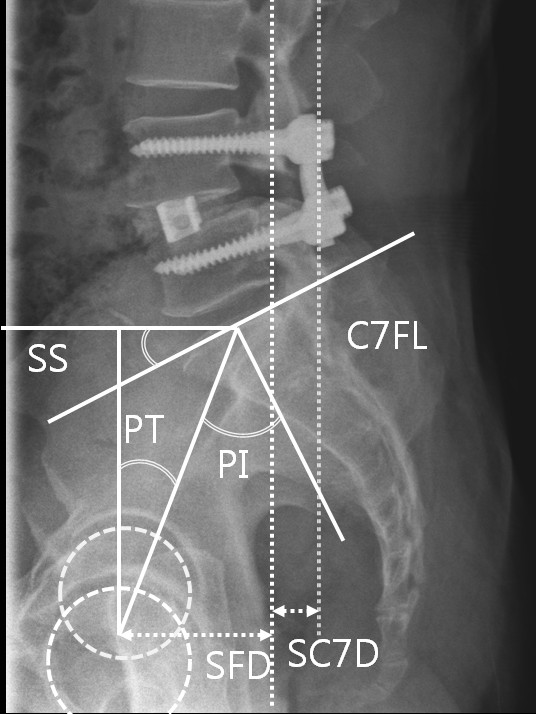
**Illustration showing the pelvic parameters included in this analysis, that is, sacral slope (SS), pelvic tilt (PT), and pelvic incidence (PI)**. PI is a morphological parameter, whereas SS and PT are positional parameters. PI represents the algebraic sum of SS and PT, (PI = SS + PT). SFD is the horizontal distance between the vertical bi-coxo-femoral axis and the vertical line passing through the posterior corner of the sacrum. The horizontal distance between C7 plumb line and the posterior corner of the sacrum (SC7D) was also measured. These two values were then used to calculate SC7D/SFD ratio, which correspond to the ratio of SC7D to SFD.

Sagittal alignments were evaluated preoperatively using 36-inch lateral films of the entire spine and both femoral heads. All films were obtained with the subject standing with arms crossed and knees fully extended with adequate lateral view of overlapping femoral heads and visualization from above the C7 vertebral body to the sacral end plate. These full spine films were evaluated for spinopelvic sagittal parameters including sacral slope(SS), PT, pelvic incidence (PI), sacrofemoral distance (SFD), and sacro-C7 plumb line distance (SC7D) (Figure 1). Other spinal parameters included in this analysis were thoracic kyphosis (TK) and lumbar lordosis (LL). The TK was measured between the upper endplate of the most inclined vertebrae into the thoracolumbar junction zone and the superior plate of C7. The LL was measured using the Cobb method between the sacral plate and the upper endplate of the most inclined vertebrae into the thoracolumbar junction zone, corresponding to the inflection point where the spine transitions from lordosis to kyphosis. Each pre, postoperative and follow up measurement for radiologic parameter was performed twice by two observers, one medical student (KMK) and one neurosurgical -spine surgeon (LSH) who are independent from the operators.

Patients were divided into two groups using improvement of PT at last follow up: group A showed a tendency towards increased and unchanged PT; and group B showed a tendency towards decreased PT to normal range. Radiologic factors and clinical outcomes were compared statistically between two groups.

### Statistic analysis

Statistical analysis was performed using PASW statistical software ver. 18.0 (SPSS Inc., Chicago, IL). The Mann-Whitney U-test was used to compare group clinical and radiological outcomes. Wilcoxon's rank sum test was used to compare differences between pre-, postoperative and final follow-up parameters of clinical and radiological outcomes. Correlation studies were performed using Pearson's coefficients to investigate relations between all radiologic parameters and VAS and ODI improvements.

## Results

Ten patients were allocated to Group A (group of the increased and unchanged PT) and eight to Group B (group of the decreased PT). Table 1 summarizes the demographic characteristics. There were 10 females and 8 males. The patients' ages ranged from 48 to 72 years (mean, 60.8 years). The mean follow-up period was 43.1 months with a minimum period of 2 years (range 24-84 months). Table 2 shows clinical values and spinopelvic parameter of each group.

**Table 2:** Result of clinical outcomes and spinopelvic parameter of 18 patients

case	VAS		ODI		Pelvic tilt			Sacral slope			Pelvic incidence			Lumbar lordosis			SC7D/SFD		
	
	preop	lastFU	preop	lastFU	preop	postop	lastFU	preop	postop	lastFU	preop	postop	lastFU	preop	postop	lastFU	preop	postop	LastFU
A1	8	2	38	6	16	21	22	28	25.4	25.2	44	46.5	46.9	47	38.2	45.4	-0.24	0.91	0.67
A2	8	7	46	36	25.3	31.6	27.1	36.2	31.2	37.7	62.3	60.5	63.8	38.7	44.2	41.3	1.96	1.35	1.08
A3	8	2	36	28	30.7	26.3	30.4	17.8	21.3	21.5	47.6	49.2	50.9	38.1	45.5	46.1	0.97	0.76	0.52
A4	3	4	36	18	19.9	25.5	26.1	42.2	30.1	28.8	62.1	55.6	54.9	52.5	61.6	62	0.50	0.30	0.56
A5	7.5	3	46	50	25.3	34.2	38.7	19.6	13.3	11.2	44.9	47.5	49.9	41.6	47.2	52.6	0.99	0.82	0.98
A6	6	7	24	12	20.9	23.1	23.6	30.6	30.8	29.6	51.5	53.9	54.2	54.1	48.9	48.1	0.01	0.54	0.82
A7	10	0	60	12	15.1	21.4	17.7	26.6	18.5	23.1	41.7	39.9	40.8	34	29.7	32.7	0.60	0.68	0.32
A8	7	2	42	32	21.4	21.8	21.1	35.3	31.3	38.2	56.7	53.1	58.3	43.5	32	47.4	1.23	1.47	0.26
A9	9	1	86	16	24.3	20.5	26.2	31	33.2	27.3	55.3	53.7	55.5	50.4	45.6	36.4	2.23	1.16	0.76
A10	2	4	18	26	16.1	18	19.3	44.8	44.2	45.9	60.9	62.2	62.2	65.2	56.8	68.7	-0.17	0.22	-0.95
B1	8	2	44	12	23.9	22.5	20	40.7	43	46.3	64.6	65.5	66.3	51.6	58.1	55.6	0.92	-0.32	0.20
B2	5	1	36	6	27.7	22.8	15.2	27.2	31.2	37.5	54.9	54	52.7	32.8	46.7	53.3	0.44	0.12	-0.10
B3	10	2	74	16	25.1	30.7	20.3	29.8	24.3	34.7	54.9	55	55	42.7	40.8	51	0.69	0.60	0.50
B4	8	2	44	8	22.4	20.2	14.7	31.5	34	38	53.9	54.1	52.7	44.4	41.4	52.9	0.18	-0.45	-0.10
B5	7	2	36	23	20	17.5	14.5	24.1	21.2	26.2	44.1	38.7	40.7	24.7	20.8	33.3	1.09	0.19	-0.25
B6	6.5	2	54	16	29.3	30.9	23.7	32.7	30.4	37.6	62	61.3	61.3	38.8	38.4	43.8	1.34	0.75	1.07
B7	5	1	88	50	31.2	26	22	20.2	26	30.1	51.4	52	52.1	37.8	31.7	41	0.29	0.27	0.29
B8	5	1	27	20	31.3	25.7	16.8	26.7	23.6	43	58	59.3	59.8	34.4	41.2	48.1	0.98	-0.27	0.53

SC7D = sacro-C7 plume line distance; SFD = sacro-femoral distance

Most patients showed improvement of symptoms related to radiculopathy immediately after surgery. Mean VAS for all patients was 6.83 (range 9.03-4.63) before surgery and this improved after surgery to 2.50 (range 0.6-4.4). The mean ODI was 46.4% (range 26.8-66%) before surgery and this too improved after surgery to 21.5% (range 18.3-24.7%). Mean VAS at last follow-up assessments were 3.20 in Group A and 1.63 in Group B; corresponding to a VAS improvement of 53.3% in Group A and of 76.1% in Group B. Mean ODI at last follow-up assessments were 43.2 in Group A and 50.4 in Group B; corresponding to an ODI improvement of 45.4% in Group A and of 61.7% in Group B. VAS and ODI improvements at follow-up were poorer in group A than in Group B (Table 3). Table 4 showed results of spinopelvic parameters in the two groups. No significant difference was found between preoperative and postoperative variables except PT in Group A. However, significant difference were found between the preoperative and postoperative values of PT, SS, TK, LL, and SFD/SC7D in Group B. Between parameters of group A and B, significant difference were found on last follow up PT. Additionally there is borderline significance on preoperative PT, preoperative LL and last follow up SS.

**Table 3:** Comparison of clinical outcomes in the two study groups based on changes in pelvic tilt after spinal fusion.

Group	VAS		Improvement rate of VAS	ODI		Improvement rate of ODI
				
	Preoperative	Last FU		Preoperative	Last FU	
A (n = 10)	6.85 ± 2.53	3.20 ± 2.35	53.3%^†^	43.2 ± 19.0	23.6 ± 13.4	45.4%^†^
B (n = 8)	6.81 ± 1.81	1.63 ± 0.52	76.1%^†^	50.4 ± 20.8	18.9 ± 13.8	61.7%^†^
*P *value*	ns	< 0.05	< 0.05	ns	< 0.05	< 0.05

* Statistical significance of value between group A and B was determined using Mann-Whitney U test.

† p < 0.05; Statistical significance of value between preoperative and last follow up was determined using Wilcoxon's signed rank test.

Values are presented as means ± standard deviations

**Table 4:** Mean values ( ± standard deviation) of Spinopelvic parameters in the two study groups based on changes in pelvic tilt after spinal fusion

Variable (normal range*)	Pelvic tilt(°) (12-18)			Sacral slope (°) (36-42)			Pelvic incidence(°) (48-55)		
	
Group	Preop	Postop	Last FU	Preop	Postop	Last FU	Preop	Postop	Last FU
A (n = 10)	21.5 ± 5.0	24.3 ± 5.1	25.1 ± 6.2^†^	31.2 ± 8.8	27.9 ± 8.7	28.9 ± 9.8	52.7 ± 7.9	52.2 ± 6.7	53.7 ± 7.0
B (n = 8)	26.4 ± 4.2	24.5 ± 4.7	18.4 ± 3.6^†^	29.1 ± 62	29.2 ± 7.1	36.7 ± 6.5^†^	55.5 ± 6.3	55.0 ± 8.0	55.1 ± 7.7

*p *value**‡**	0.06	ns	0.01	ns	ns	0.09	ns	ns	ns

**Variable** (normal range*)	**Thoracic kyphosis(°)** **(41-48)**			**Lumbar lordosis(°)** **(43-61)**			**SC7D/SFD** **(-1.9-0.1)**		
	
**Group**	**Preop**	**Postop**	**Last FU**	**Preop**	**Postop**	**Last FU**	**Preop**	**Postop**	**Last FU**

A (n = 10)	38.4 ± 9.7	36.4 ± 8.8	33.7 ± 10.0	46.5 ± 9.3	45.0 ± 9.9	48.1 ± 10.9	0.81 ± 0.9	0.82 ± 0.4	0.50 ± 0.6
B (n = 8)	35.2 ± 8.9	37.1 ± 8.9	41.3 ± 9.6^†^	38.4 ± 8.1	39.9 ± 10.8	47.4 ± 7.5^†^	0.74 ± 0.4	0.11 ± 0.4	0.27 ± 0.4^†^

*p *value**‡**	ns	ns	ns	0.09	ns	ns	ns	0.04	ns

SC7D = sacro-C7 plume line distance; SFD = sacro-femoral distance

*Values are means from studies that included adult subjects aged 20 to 85 years without any symptom or history suggestive of spinal disease [5,7,22-33].

†p < 0.05; Statistical significance of value between preoperative and last follow up was determined using Wilcoxon's signed rank test.

**‡**p < 0.05; Statistical significance of value between group A and B was determined using Mann-Whitney U test.

Correlation analysis between all radiologic parameters of sagittal balance and VAS and ODI improvement in Groups A and B are presented in Table 5. VAS improvements in Group A were found to be significantly related to postoperative lumbar lordosis (Pearson's coefficient = -0.829; p = 0.003). Similarly, ODI improvements were also found to be significantly associated with postoperative lumbar lordosis (Pearson's coefficient = -0.700; p = 0.024). However, in Group B, VAS and ODI improvements were not found to be related to postoperative lumbar lordosis and to spinopelvic parameters.

**Table 5:** Pearson's correlation coefficients between spinopelvic and clinical parameters in the two study groups

Parameter	Group A (n = 10)		Group B (n = 8)	
	
	△VAS	△ODI	△VAS	△ODI
Sacral slope (°)	-0.612	-0.327	0.128	0.281
Pelvic incidence(°)	-0.621	-0.560	0.051	0.270
Thoracic kyphosis(°)	-0.096	-0.265	0.105	0.251
Lumbar lordosis(°)	-0.829^†^	-0.700*	0.394	0.675
SC7D/SFD	0.519	0.493	-0.152	-0.025

SC7D = sacro-C7 plume line distance; SFD = sacro-femoral distance

△VAS = Improvement rate between preoperative VAS and Last follow up VAS; △ODI = Improvement rate between preoperative ODI and Last follow up ODI

*P < 0.05, or †P < 0.01

## Discussion

The optimal surgical approach to the management of lumbar degenerative spondylolisthesis has yet to be determined. Although gross spinal imbalance in association with degenerative lumbar spondylolisthesis is rare, more subtle forms of segmental imbalance may influence early surgical outcome and the later development of adjacent segment disease [3,13,14]. Furthermore, although slippage and lordosis at the level of spondylolisthesis have been evaluated radiologically, few reports have discussed lumbar sagittal balance in patients with degenerative lumbar spondylolisthesis

In an early study on 95 patients, Schwab *et al *identified radiologic parameters correlated with self-perceived pain (measured using a VAS scale), namely, intervertebral subluxation (olisthesis), L3 and L4 coronal vertebral obliquity, and loss of lumbar lordosis [15]. In addition, in a later report, loss of lordosis was also found to be correlated with lower Short Form 36 (SF-36) scores [16]. More recently, Glassman et al investigated the relationship between global alignment and measures of HRQOL, and found that sagittal vertical axis (measured as the offset between C7 plumb line and the posterosuperior corner of S1) was correlated with pain and a decrease in function as measured using ODI and SF-12 [3,4]. Considerable work is now directed at improving our understanding of not only ideal spinal alignment but also of spinopelvic relationships. Lazennec et al found that PT was correlated with increased pain in patients that underwent a lumbosacral fusion and demonstrated that patients with a larger postoperative PT were more likely to demonstrate residual pain [17].

### Clinical and radiological outcomes based on postoperative PT

We noted that a change of pelvic tilt after spinal fusion was associated with improvements in VAS and ODI, and a statistically significant association was observed between lumbar lordosis and improvements in VAS and ODI in patients whose postoperative pelvic tilt was not improved. By classifying patients according to an in improvement in pelvic tilt after operation demonstrated that pain and disability were greater in Group A than in Group B. Furthermore, patients in Group A had higher levels of self-reported pain and disability. In other words, patients with a non-improved PT postoperatively also had lower HRQOLs. In Group A, patients showed a tendency toward a poor clinical outcome. However, interestingly patients who presented with lower LL showed a better clinical outcome, and patients without lower LL in Group A experienced the highest levels of self reported pain and disability. On the other hand, in Group B, patients had better clinical outcomes regardless of LL values and the other parameters.

Actually, it would seem that there is the morphological difference between both groups preoperatively although this difference is not significant statistically. In group B with lumbar hypolordosis, operation could improve the lumbar lordosis into normal range by compression following decreasing PT. In group A with well compensated spondylolisthesis showing normal lumbar lordosis, however, operation could not improve any spinopelvic parameter including lumbar lordosis and pelvic tilt. Finally, the surgical result of group with lumbar hypolordosis was better than those of group with normal lumbar lordosis.

### Importance of PT and lumbar lordosis

Improvements in PT postoperatively played a significant role in the achievement of a good clinical outcome, as determined by VAS and ODI. Although postoperative PT was not improved, LL also contributed to a good clinical outcome. It is becoming increasingly recognized that studies of spinal alignment should include pelvic position. Although PI determines LL, PT is a positional parameter that reflects compensation to spinal deformity. This study confirms that pelvic position, as measured by PT, is correlated with HRQOL in the setting of adult deformity, and that high PT values reflect compensatory pelvic retroversion due to sagittal spinal malalignment. Furthermore, the significant correlation found between postoperative PT improvement and clinical outcome appears to indicate that resetting PT to a normal value is important for restoring ambulatory function by improving the range of hip extension.

However, Increasing PT is not the only way to compensate a local loss of lordosis. The primary compensation is obtained by hyper extension of the segment above or below the hypolordosis area. This study also confirms the presence of relationships between LL and clinical outcomes, which is consistent with published reports [18,19]. LL is a key consideration when analyzing radiographic alignment, and is thought to cause chronic lower back pain by increasing traction loadings on posterior elements of the spine, including the paravertebral muscles [20].

**Figure 2:**
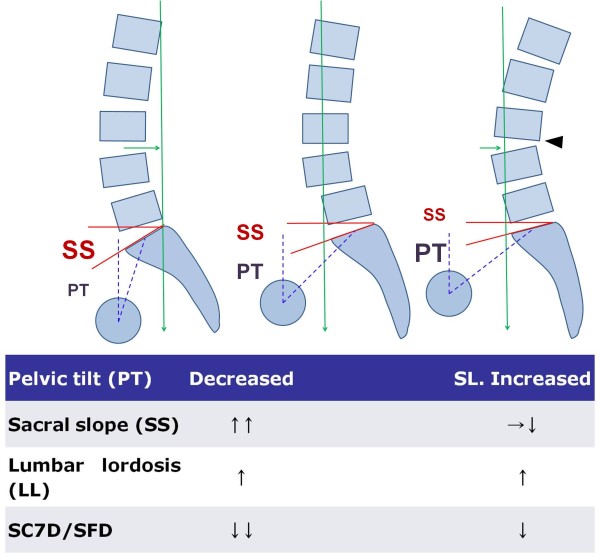
**Patients with degenerative spondylolisthesis (DSPL) are characterized by an increased pelvic tilt (PT) and decreased sacral slope (SS) than the control population, suggesting the presence of a pelvic compensation (center)**. Additionally, Disc degeneration and slippage at the DSPL level led to an anterior sagittal imbalance with anterior displacement of the C7 plumb line (green arrows). In the DSPL patients achieved optimal lumbar lordosis (LL) after surgery, the pelvis backtilt (decreased PT) and anterior displacement of the axis of gravity is improved (left). In the DSPL patients of hypolordotic reduction, we suppose that the aggravating pelvis backtilt (more increased PT) permits the limiting of the anterior displacement of the axis of gravity as well as hyperextension and retrolisthesis (arrow head) observed in the upper lumbar spine (right).

Therefore, obtaining lordosis at fused segments may be useful for controlling lower back pain. In case of a hypolordotic fusion, there is a painful compensation in hyper extension at the upper level. The global LL does not change but the patient is not improved. PT remains slightly impaired, but the pain is due to the hyper extension compensation (Figure 2). Such residual lower back pain may result from postoperative change of the zygapophysial joint, the paravertebral muscle, the posterior ligamentous complex and the adjacent segment change.

### Other Radiographic Parameters of Importance

The issue of spinal balance is attracting attention in the literature. However, conflicting results have been reported about the relationship between radiological factors related to sagittal balance and clinical outcomes after lumbar arthrodesis [10,21]. With regard to early clinical outcomes, Kawakami et al. noted improved clinical recovery rates in patients that underwent fusion for degenerative spondylolisthesis when the L1 axis S1 interval (their measure of the position of the plumb line in front of the sacrum) was less than 35 mm and when lordosis of the fused segments was achieved [10]. However, although the correction of sagittal plane deformity may be achieved by a number of means, there appears to be relatively little information in the literature regarding degenerative spondylolisthesis. In the present study, no significant relationship was found between radiological factors related to sagittal balance and clinical success except PT and LL.

## Conclusion

This is the first study to evaluate the impact of sagittal balance in patients with degenerative spondylolisthesis that underwent fusion surgery. Thus, clinically, analyses of spinopelvic parameters, such as, pelvic tilt and lumbar lordosis, appear to be essential to the understanding of the impact of spinal deformity and the treatment choice in degenerative lumbar spondylolisthesis. Nevertheless, the retrospective study design of the present study and the small number of patients included should be considered when interpreting our results.

## Competing interests

The authors declare that they have no competing interests.

## Authors' contributions

MKK collected data and drafted the manuscript. SHL has made substantial contributions to conception, design, data collection and revised it critically for important intellectual content. ESK, WE, SSC participated in analysis and interpretation of data, CSL participated in its design. All authors gave final approval of the version to be published.

## Pre-publication history

The pre-publication history for this paper can be accessed here:

http://www.biomedcentral.com/1471-2474/12/69/prepub
